# An Overview of Circular RNAs and Their Implications in Myotonic Dystrophy

**DOI:** 10.3390/ijms20184385

**Published:** 2019-09-06

**Authors:** Karol Czubak, Saam Sedehizadeh, Piotr Kozlowski, Marzena Wojciechowska

**Affiliations:** 1 Department of Molecular Genetics, Institute of Bioorganic Chemistry, Polish Academy of Sciences, 61-704 Poznan, Poland; 2 Queen’s Medical Centre, School of Life Sciences, University of Nottingham, Nottingham NG7 2QL, UK

**Keywords:** circular RNAs (circRNAs), back-splicing, myotonic dystrophy type 1 (DM1), alternative pre-mRNA splicing, muscleblind-like proteins, RNA-binding proteins

## Abstract

Circular RNAs (circRNAs) are a class of single-stranded covalently closed RNA rings. Biogenesis of circRNAs, which may occur co-transcriptionally and post-transcriptionally via a back-splicing mechanism, requires the presence of complementary and/or inverted repeat sequences in introns flanking back-spliced exons and is facilitated by RNA-binding proteins. CircRNAs are abundant across eukaryotes; however, their biological functions remain largely speculative. Recently, they have been emerging as new members of a gene regulatory network and contributing factors in various human diseases including cancer, neurological, muscular and cardiovascular disorders. In this review, we present an overview of the current knowledge about circRNAs biogenesis and their aberrant expression in various human disorders. In particular, we focus on the latest discovery of circRNAs global upregulation in myotonic dystrophy type 1 (DM1) skeletal muscles and the role these prospective biomarkers might have for prognosis and therapeutic response in DM1.

## 1. Introduction

Circular RNAs (circRNAs) were originally identified in the late 1970s in pathogens, including viroids (virus-like infectious particles) [1]. A handful of circRNAs generated in eukaryotic cells were subsequently identified in the 1990s, but at that time, the molecules did not gain much interest and were considered byproducts of aberrant RNA splicing as they were generally detected at low levels (~0.01% the level of their associated linear mRNA) [2,3,4]. However, with the advent of high-throughput RNA sequencing (RNA-Seq) and novel bioinformatics tools and databases recent experimental data showed that circRNAs are ubiquitous molecules expressed across eukaryotes including human, mice, *D. melanogaster*, *C. elegans*, *S. pombe* and plants [5,6,7,8,9]. Thousands of protein-coding genes generate circRNAs, and although the levels of most circRNAs are low, for some genes (e.g., *SMARCA5*, *UBXN7* and *PNN*), their abundance exceeds that of the associated linear mRNAs [10,11].

CircRNAs are covalently closed rings with no 5′-cap and 3′-poly (A) termini. Perhaps, such a unique structure determines high stability of these molecules which unlike most of other types of RNAs (i.e., mRNAs, tRNAs, 5S RNA) are resistant to the 3′→5′ exoribonuclease digestion with RNase R [12]. To date, very little is known about mechanisms of circRNAs turnover and studies suggested that they may be globally degraded by RNase L [13,14] or eliminated from cells via exosomes [15]. Most of these highly stable transcripts are encoded by protein-coding genes, derive from their exons (E/circRNAs) and accumulate in the cytoplasm, but do not generally associate with ribosomes [16]. However, there are also circRNA species that contain both exonic and intronic sequences (E-I/circRNAs), as well as circRNAs derived exclusively from introns (I/circRNAs) of protein coding genes. CircRNAs can be formed both co-transcriptionally and post-transcriptionally in a process known as back-splicing or head-to-tail circle splicing, which involves joining of a splice donor to an upstream splice acceptor [17,18]. Both computational analyses of RNA-Seq data and plasmid-based experiments have pointed to repetitive elements (complementary sequences and/or inverted Alu repeats) in introns as critical regulatory sequences that promote most backsplicing events [5,19,20,21,22,23]. Moreover, some of the *trans*-acting factors that collaborate with the repeats are beginning to be identified, among which are RNA-binding proteins (RBPs) either promoting or impeding the circularization process [5,8,9,24,25,26]. There are reported examples that the generation of circRNAs employs canonical splicing machinery [22]. CircRNAs can also interact with microRNAs (miRNAs) and act as decoys for specific miRNAs preventing them from inhibiting target RNAs and thereby post-transcriptionally regulate expression of the parental and other genes [7,27,28,29,30]. However, while only three circRNAs are known as efficient sponges for miRNAs, most others contain none or few miRNA binding sites and likely have a different function [7,31,32].

The biology and function of most circRNAs are still poorly recognized. Recent rapid interest in this class of molecules has resulted in considering circRNAs as a group of physiological RNA molecules with an important role in normal cell biology, and circRNAs dysregulation has been reported to be associated with human diseases. The expression of circRNAs has recently been reported to generally decrease in human cancers [33,34,35] and increase in some late-onset human neurological and muscular disorders [9,15,36,37,38,39,40] suggesting that their expression can be drastically altered and potentially contribute to pathological states. There are reports suggesting circRNA involvement in cardiovascular and autoimmune conditions [41,42,43,44,45,46]. CircRNA functions may also be related to their elevated stability that is higher than their linear counterpart mRNAs, which may reflect the fact that these small molecules have been found to accumulate with age especially in tissues of low proliferative capacity. Notably, circRNA abundance tends to increase during the differentiation process, and they were found to be highly expressed in mammalian brain and striated muscle tissues (both skeletal and cardiac) and they are one of the body tissues showing the highest levels of alternative splicing. Lately, an elevated pattern of expression of circRNAs was documented in skeletal muscles of patients suffering from Duchenne muscular dystrophy (DMD) and myotonic dystrophy type 1 (DM1) [36,40,47,48]. In both the diseases the differential expression of circRNAs levels was correlated with aberrant alternative splicing.

DM1 is the most common form of adult-onset muscular dystrophy, which is characterized by progressive muscle wasting and weakness. This autosomal dominant disorder is caused by an expansion of CTG repeats in the 3′-UTR of the *DMPK* gene [49,50]. In DM1 patients, the CTG repeats are expanded to hundreds and thousands of copies, whereas non-DM1 individuals have below 37 CTG repeats. DM1 pathogenesis is associated with gain-of-function mechanism of toxic mutant DMPK mRNA (rCUGexp) which accumulates in punctate nuclear foci [51]. Their presence is correlated with the sequestration of muscleblind-like (MBNL)-family proteins leading to the functional insufficiency of these splicing factors. Another consequence of expression of rCUGexp is hyperphoshorylation of CUGBP1 protein causing its longer half-life and increased levels in DM1 [52,53]. One of the molecular consequences of the imbalance in MBNL and CUGBP1 proteins is aberrant alternative splicing of many target genes found in patients which represents a basis of DM1 pathomechanism. For example, mis-splicing of the *CLCN1* exon 7, the *INSR* exon 11, and the *BIN1* exon 11 were shown to be associated with reduced chloride conductance, lower insulin responsiveness, and muscle weakness, respectively [54,55,56,57]. However, nuclear deposition of rCUGexp leads to abnormalities in many pathways of RNA metabolism [58] and recently discovered dysregulation of circRNAs in DM1 muscles may be one of them [36,40].

In this review, we describe and discuss the most current knowledge about circRNAs biogenesis, regulations and functions. We present an overview of circRNAs aberrant expression and prospective involvement in various human disorders. In particular, we focus on the most recent findings in this field and discuss global upregulation of circRNAs in DM1 skeletal muscles and the role of these potential biomarkers for prognosis and therapeutic response in DM1.

## 2. Biogenesis of CircRNAs

### 2.1. Repeat Sequences in CircRNAs Biogenesis

**Figure 1:**
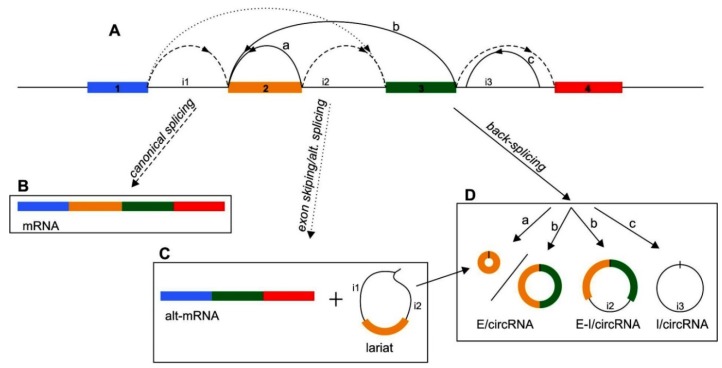
Schematic presentation of circular RNAs (circRNAs) biogenesis. (**A**) Schematic representation of pre-mRNA with indicated exons (colored rectangles), introns (named as i1, i2 and i3), and different forms of splicing. Pre-mRNA undergoes a canonical (**B**) and non-canonical (**C** and **D**) splicing. (**B**) In a canonical splicing (dashed lines in panel **A**) consecutive exons are joined together (i.e., exon 1 to exon 2 to exon 3, etc.) to generate a linear mRNA that is subsequently translated. (**C** and **D**) Pre-mRNA may also be processed in a non-canonical splicing via either exon skipping (**C**), (dotted lines in panel **A**) or via back-splicing (**D**), (solid lines in panel **A**). Exon skipping events can lead to the generation of a linear mRNA when, e.g., exon 1 is joined directly to exon 3. It is worth noting that lariat is a byproduct of exon skipping, and may facilitate back-splicing, which eventually gives rise to a circRNA composed of the skipped exon (e.g., exon 2) (**C**). Different types of circRNAs (**D**), i.e., exonic (E/circRNAs), exonic-intronic (E-I/circRNA) and intronic (I/circRNA) are generated through back-splicing of the 5′ splice donor to the 3′ splice acceptor (e.g., joining the 3′ end of exon 3 to the 5′ end of exon 2). E/circRNAs may be generated from: (a) a single exon (e.g., exon 2), or from (b) two or more exons (e.g., exons 2 and 3). The E-I/circRNAs (b) may be generated from retaining intron which separates exons participating in circRNA formation (e.g., exons 2 and 3, and intron 2). However, the I/circRNAs (c) may be generated through interactions of reverse complementary sequences present in lariat introns (e.g., intron 3) excised from pre-mRNA.

Nearly all genes are subjected to alternative splicing events and canonical pre-mRNA splicing can be accompanied by non-canonical splicing which includes an exon skipping mechanism and circRNAs biogenesis [32,59]. Different linear and circRNAs can be produced depending on how a pre-mRNA is spliced (Figure 1A–D). In the standard way of splicing (i.e., exon 1 is joined to exon 2, which is joined to exon 3, etc.), a linear mRNA is generated and subsequently can be translated to produce a protein (Figure 1A,B). However, it remains unknown how the splicing machinery decides whether to generate a linear mRNA or a circRNA and the precise mechanism of circRNAs biogenesis remains unknown. It has been suggested that the process in which pre-mRNA produces a circular RNA involves a head-to-tail (back-splicing) linking process in which a splice donor is joined to an upstream splice acceptor (e.g., the end of exon 3 is joined to the beginning of exon 2) (Figure 1A,D). In most cases, these scrambled transcripts are noncoding as the start and/or stop codons have been removed. The back-splicing can also be combined with exon skipping so that a linear mRNA as well as a circRNA comprised of the skipped exon(s) can be generated from a single pre-mRNA (Figure 1A,C).

Bringing back-splice sites in close proximity is facilitated by the interactions between introns which flank circRNA-forming exons. In particular, the presence of repeat sequences (paired Alu-repeats in inverted orientation) in the introns can strongly promote circularization. A connection between repetitive elements and circRNAs biogenesis was first suggested at the mouse *Sry* locus, which produces a ~1.2-kb long circRNA, comprised of a single exon [60]. Remarkably, this exon is flanked by very long (~15,000 nt) of almost perfectly complementary intronic sequences, and as shown in in vitro plasmid experiment removal of the whole repeats eliminated Sry circRNA production [61]. However, repeats as long as those at the *Sry* locus do not often flank exons naturally. However, in silico analysis revealed that Alu elements, which are >300-nt long, are enriched in the introns flanking human exons that generate circRNAs [20,21,60], and almost 90% of circRNAs appeared to have complementary Alu elements in their flanking introns [5,21]. Therefore, these data suggest that the Sry circRNA biogenesis model is likely applicable at thousands of human genes, and shorter stretches of base pairing between intronic repeats appeared often to be sufficient [19]. Importantly, this mechanism seems to be used across eukaryotes and inverted repeats were found to flank circRNAs in mice and *C. elegans* [21]. However, there are some exceptions and the introns that border many *D. melanogaster* circRNAs do not contain complementary sequences [62]. Instead, a positive correlation between the length of the flanking introns and circRNAs abundance was identified in *D. melanogaster*. This suggests that circRNA biogenesis in flies may occur via a distinct mechanism, e.g., some non-complementary interactions between introns and the binding of splicing factors to both flanking introns, as has been proposed at the muscleblind (*mbl)* locus in *D. melanogaster* [8]. Interactions between proteins that bind two separate introns could promote backsplicing similarly to how base pairing between intronic repeats is thought to bring the intervening splice sites into close proximity. However, cells seem to generate a variety of other circles through unique strategies; for instance, a distinct class of circRNAs is known to be generated from the introns of some protein-coding genes when the introns do not undergo debranching process [63].

It seems that only a small subset of exons undergo back-splicing generating detectable amounts of circRNAs, although most internal exons in multi-exon genes can theoretically self-circularize as they have splicing signals at the 5′ and 3′ ends. Thus, the question is: what mechanism/factors control the circularization? Additionally, introns normally contain multiple repetitive elements and depending on how the complementary sequences base pair to one another, very different splicing patterns can result (Figure 1D). Thus, what mechanism/factors control the expression of a given circRNA? One could speculate that this is dependent on the competition for base pairing between the various complementary sequences, the number of repeats, their degree of homology and the distance between them are likely all key determining factors [64]. Thus, there must exist a multi-layered regulatory system in the selection of circRNA exons conducting the process of circularization. Since circRNAs biogenesis can occur both co-transcriptionally and post-transcriptionally, a variety of RBPs including splicing factors must be engaged in the process of circRNAs generation. Thus far, a small group of RBPs has been found to impact back-splicing, among which are proteins either promoting or impeding a circularization process [8,9,25,26].

### 2.2. Proteins of CircRNAs Biogenesis

**Figure 2:**
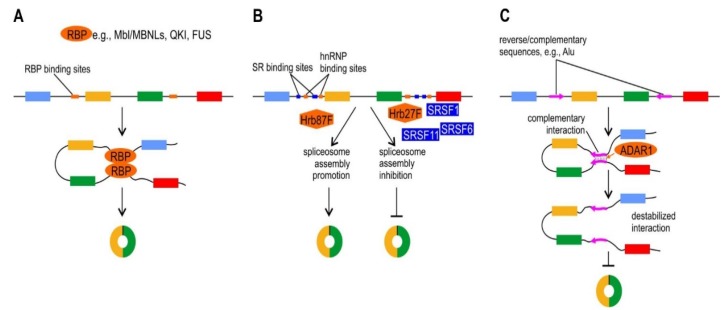
Proteins involved in circRNA biogenesis and potential mechanism of their actions. Exons are represented by rectangles, as in Figure 1, the “T” bars indicate inhibition of circRNA formation. (**A**) Mbl/MBNLs, Quaking (QKI) and Fused in Sarcoma (FUS) proteins can promote circRNAs biogenesis via binding in their RNA-binding motifs in introns adjacent to the back-splicing junctions facilitating the bridging process. (**B**) hnRNPs (heterogeneous nuclear ribonucleoproteins) and SR (serine-arginine) proteins can act in a combinatorial manner to either stimulate or inhibit circRNAs formation. Depletion of SR proteins (SRSF1, SRSF11 and SRSF6) each can cause an increase of circRNAs level. Similarly, hnRNP protein Hrb27C acts to impede circRNAs generation, whereas Hrb87F can enhance the process. In addition, simultaneous depletion of Hrb27C with SRSF1, SRSF11 and SRSF6 cause an additive effect and increase of circRNAs expression, suggesting that each of these factors plays a non-redundant role. (**C**) ADAR1 protein hampers circRNAs biogenesis via destabilizing double-stranded RNA interactions of introns flanking circRNA-forming exons through site-specific deamination of adenosine bases to inosines. ADAR1 interferes with RNA-RNA interactions between inverted repeats present in circRNA-flanking introns (shown as pink arrows) and prevents the formation of structures promoting circularization of exons.

To date, several *trans*-acting factors have been proposed to participate in circRNAs biogenesis. Most of them are known splicing factors; however, the role of other RBPs cannot be excluded. The very first RBP proposed to take part in circRNAs biogenesis was *D. melanogaster* Mbl splicing factor, an ortholog of human MBNL-family proteins (Figure 2A). Members of the muscleblind proteins in *D. melanogaster* and mammals are conserved RNA-binding factors and key players of alternative splicing regulation of hundreds of pre-mRNAs during development [65,66]. Despite this primary function, MBNL proteins also regulate subcellular localization and stability of mRNAs and alternative polyadenylation [58]. In human, MBNL-family proteins consist of three paralogs, namely MBNL1, MBNL2 and MBNL3, which are critical for skeletal, cardiac and nervous system functions [67]. The proteins contain four zinc fingers serving as RNA-binding domains, which specifically recognize an RNA motif consisting of a GpC dinucleotide flanked by pyrimidines, YGCY (Y–U or C). Functionally, the family has been linked to splicing aberrations characteristic of human disease DM1 [65,67,68,69]. It was experimentally shown in a plasmid system that the generation of a circRNA derived from the second exon of the *Mbl* gene (circMbl; dme_circ_0001678) was regulated by the Mbl protein itself. The mechanism of this regulation was associated with the presence of Mbl-binding motifs in introns flanking circMbl [8]. In both human and *D. melanogaster* the exogenous expression of Mbl protein stimulated circRNA production from endogenous MBNL1/Mbl transcripts. Moreover, downregulation of Mbl protein in the fruit fly cell culture and neural tissues resulted in decrease in circMbl level. Interestingly, the elevation of Mbl was found to increase not only circMbl, but also the levels of several other circRNAs, suggesting a more general role of the protein in circRNA biogenesis. However, most recent results from two research groups [36,40], which used DM1 muscle biopsy tissues as a natural model of MBNLs deficiency, showed instead a global elevation of circRNA levels. This discovery questions the original idea of MBNLs being universal factors of circRNAs biogenesis, and suggests that the proteins, rather than playing a global regulatory role, facilitate the formation of individual species of circRNAs.

Quaking (QKI) is another protein which promotes circRNAs production (Figure 2A). The QKI, which belongs to the STAR family of KH domain-containing RBPs, has been found to affect pre-mRNA splicing [70,71], mRNA turnover [72] and translation [73] and is implicated in diseases including ataxia, schizophrenia and glioblastoma [74]. Recently, as shown by Conn et al. [24], QKI can promote circRNAs biogenesis during epithelial to mesenchymal transition (EMT). The EMT is a cellular differentiation process important in embryo development, wound healing and in cancer metastasis [75]. The process involves changes in cell morphology and in gene expression patterns, and can be triggered by various ligand-receptor interactions, including TGF-b [76]. Conn et al., [24] reported that during the EMT, expression of hundreds of circRNAs was altered in response to TGF-b, with the majority of circRNAs increasing in abundance. The QKI protein was a major regulator of circRNAs biogenesis in EMT. This regulation was dependent on the presence of consensus binding sequences for QKI (5’-NACUAAY-N(1,20)-UAAY-3’) in circRNA-flanking introns. This observation is consistent with an earlier photoactivatable ribonucleoside enhanced cross-linking and immunoprecipitation (PAR-CLIP) results in human embryonic kidney cells (HEK293T), indicating that the majority of QKI binding occurs within its intronic binding motifs, supporting the QKI role in splicing [77]. The insertion of QKI binding motifs into the flanking introns induced circRNAs formation from exons that undergo only canonical linear splicing [24]. Since QKI is a dimer and can bind two distant regions of a single RNA molecule [78], it is feasible that the protein similarly promotes circRNA biogenesis by bringing the circle-forming exons into close proximity.

The back-splicing reactions leading to circRNAs production were also shown to be regulated by FUS (Fused in Sarcoma) protein (Figure 2A). FUS is a multifunctional protein and has been reported to play a role in various aspects of RNA metabolism, including splicing regulation [79]. The protein has been of medical interest because point mutations in *FUS* can cause neurodegenerative disorders such as amyotrophic lateral sclerosis (ALS) and frontotemporal dementia (FTD) [80]. Most FUS mutations linked to ALS are clustered in the C-terminal part of the protein in or near the nuclear localization signal. This leads to the aberrant localization of the protein in the cytoplasm, its decrease in the nucleus and formation of abnormal cytoplasmic aggregates [80,81,82]. It has been proposed that aberrant RNA metabolism due to FUS mutations by gain- and/or loss-of function is key mechanism in the pathogenesis of ALS and FTD [83]. Recent results suggest that the production of circRNAs expressed in in vitro-derived mouse motor neurons (MNs) was regulated by FUS. Errichelli et al. [25] showed that the levels of a subgroup of circRNAs were downregulated by depletion of FUS and by mutations in *FUS* linked with familial forms of ALS. Remarkably, for the majority of the dysregulated circRNAs, changes were not correlated with their linear counterparts. Particularly, most of the circRNAs altered upon FUS reduction in mouse MNs were conserved in human iPSCs-derived MNs, suggesting that the activities of this novel class of transcripts may be conserved as well. Inversely, an elevation of circRNAs was found when FUS was ectopically expressed, indicating a direct relationship between the amount of back-splicing and the levels of FUS. The PAR-CLIP results showed that for the selected circRNAs that were FUS-regulated, the enrichment of FUS binding sequence 5′-GUGGU-3′ [84] in introns proximal to the back-splicing junctions facilitated circRNAs production, suggesting a direct role of the protein in back-splicing regulation. This is in agreement with earlier work showing that FUS preferentially binds the 5′ end of very long introns and, in particular, around the alternatively spliced exons of genes associated with neuronal functions and neurodegeneration [84,85,86].

RNA-binding motif protein 20 (RBM20) is yet another RBP involved in an RNA circularization process [9]. RBM20 acts as a regulator of mRNA alternative splicing of a subset of genes involved in cardiac development. One of its gene targets is *titin* (*TTN*), a large gene containing >350 exons (the highest number in human), which is known to undergo highly complex alternative splicing. In particular, RBM20 is responsible for alternative splicing within the I-band of TTN pre-mRNA (i.e., TTN central region with the most frequent events of differential alternative splicing), and mutations in the protein have been shown to result in the expression of large and highly compliant TTN isoforms, suggested to cause dilated cardiomyopathy [87]. Strikingly, the I-band of *TTN* has been recently found to be a hotspot of circRNAs as reported by Khan et al. [9], who found 80 different circRNAs among about a thousand from human hearts. Interestingly, this subset of circRNAs had an enrichment of RBM20 binding sites in the introns flanking the back-spliced junctions. There was a five-fold increase in RBM20 binding site frequency in the introns flanking the 80 TTN circRNAs compared to a control set of introns. The involvement of RBP20 in biogenesis of these TTN circRNAs was confirmed in the *Rbm20* null mice that completely lacked these molecules, and in a cardiac sample from a heterozygous *RBM20* mutation carrier in which production of TTN circRNAs was severely affected [9]. Interestingly, a high number of circRNAs (~100) originated predominantly from the I-band of *TTN* was also detected in human skeletal muscles from DM1 patients as reported most recently by Czubak et al., [40].

A potential role in biogenesis of circRNAs in *D. melanogaster* has been shown for hnRNPs (heterogeneous nuclear ribonucleoproteins) and SR (serine-arginine) family proteins of known splicing factors [26] (Figure 2B). It has been speculated whether the fruit fly uses unique mechanisms to determine exons to be back-spliced, since biogenesis of circRNAs in *D. melanogaster* was suggested not to be driven by base-pair interactions of repeat sequences in introns flanking back-spliced exons [62]. This issue was addressed by Kramer et al. [26], who found a positive correlation between the length of flanking introns and circRNAs abundance. The authors showed that in *D. melanogaster* circularization events of *Laccase2* gene, which is a host gene of abundant circRNAs, in vitro and in vivo was regulated by both intronic repeats and *trans*-acting splicing factors. Unlike the Mbl circRNA, which requires the Mbl protein for its biogenesis, Laccase2 circRNA (dme_circ_0001871) level was not controlled by the *Laccase2* gene product, but rather by multiple hnRNPs and SR proteins acting in a combinatorial manner. Kramer et al. [26] found that depletion of three SR proteins (SRSF1, SRSF11 and SRSF6) caused significant increase of Laccase2 circRNA level, indicating an inhibitory role for these proteins in circRNAs biogenesis. However, the hnRNP proteins Hrb87F and Hrb27C act, respectively, to enhance and impede Laccase2 circRNA generation (Figure 2B). In contrast, the level of the Mbl circRNA was largely unaffected by modulating the expression of these hnRNPs and SR proteins, indicating that distinct sets of factors regulate circularization of the Mbl and Laccase2 circRNAs. In addition, hnRNPs and SR proteins were suggested to have a combinatorial control on circRNAs biogenesis. As experimentally proven, simultaneous depletion of Hrb27C with SRSF1, SRSF11 and SRSF6 caused an additive increase in Laccase2 circRNA expression, suggesting that each of these factors plays a non-redundant role [26]. Similarly, expression of another *D. melanogaster* circRNA, PlexA (dme_circ_0001671), was found to be regulated in a combinatorial manner by hnRNPs and SR proteins [26]. Altogether, these results suggest that such multi-protein control may be a potential regulatory strategy that modulates circular RNA levels.

The RNA-editing factor ADAR1 (adenosine deaminase acting on RNA) binds double-stranded RNA and converts adenosines to inosines (A-to-I). This enzyme was recently shown to hamper circRNAs biogenesis as reported by Ivanov et al. [5]. The authors found that in *C. elegance* and in human reverse complementary sequences (i.e., inverted repeats) between introns bracketing circRNA exons were significantly enriched in comparison to the non-circularized exons. The interactions between those inverted repeats promoted formation of double-stranded RNA hairpin structures which in turn induced ‘‘head-to-tail’’ splicing, thereby inducing circRNA generation (Figure 2C). These RNA-RNA interactions between inverted repeats were antagonized by ADAR1 leading to destabilization of double-stranded RNA structures (by melting stems within these interactions and thus hampering circRNAs biogenesis). The mechanism of this melting was connected with deamination of adenosine bases to inosines, and thus dysregulation of the interactions between repeat elements present in circRNA-flanking introns. In fact, as reported by Ivanov et al. [5], introns bracketing circRNAs were highly enriched in A-to-I RNA editing events. Consequently, knockdown of this RNA-editing enzyme in *C. elegans* caused upregulation of circRNAs expression. Overall, this analysis supports a model that links circRNAs biogenesis to inverted repeats and these to hairpin formation which eventually triggers RNA editing via ADAR1. Importantly, this specific model described by Ivanov et al., [5] in *C. elegans* and human seems to be conserved across animals, since Alu repeats have expanded relatively recently in vertebrate evolution.

## 3. Biological Functions of CircRNAs and Their Involvement in Human Disorders

CircRNAs are a widespread form of small RNAs with little or no protein-coding capacity. Regardless of circRNAs ubiquitous nature it is unclear whether they are produced in a controlled manner to carry out specific cellular functions or whether they represent splicing by-products without function. The former option is supported (i) by data indicating that the patterns of circRNAs expression is developmentally regulated, tissue and cell-type specific, suggesting their formation may be controlled, which in turn would indicate they have biological functions [15], and (ii) by the fact that some circRNAs are predominant (e.g., circHIPK3; hsa_circ_0000284) or even the only product (e.g., circCDR1as; hsa_circ_0001946) of their precursor transcripts [88]. It has been proposed that circRNAs may serve as regulatory RNAs. This unique class of RNAs is suggested to alter the level of free miRNAs acting as endogenous miRNA inhibitors or sponges to quench their normal functions [27,28,30,89]. However, there are only a few well-documented circRNAs known for their sponging activities that have multiple binding sites for miRNAs. These are circCDR1as, having over 70 binding sites for miR-7, circSry, having 16 binding sites for miR138, and circHIPK3, known to have 18 potential binding sites sponging nine different miRNAs [7,31,60]. However, the majority of circRNAs contain only a few or even single potential miRNA-binding sites and appear to have different functions [16,24]. These putative functions may be correlated with the fact that circRNAs have been recognized as new members of gene regulatory networks and contributing factors in the modulation of transcription, the interference with splicing and translation of small proteins. Recent extensive interest in this class of molecules resulted in various reports of dysregulation of circRNAs expression emerging them as likely players in various human diseases including different cancers, cardiovascular diseases and autoimmune conditions, as well as neurological and muscular disorders [9,34,36,38,39,41]. Due to circRNA’s high stability, they are believed to accumulate over time in non-dividing cells and via their interactions with other RNAs and proteins may contribute to some diseases, particularly those associated with aging. CircRNAs involvement in human neurodegenerative disorders of late onset has been investigated, and for example in the Alzheimer’s disorder (AD), the downregulation of circCDR1as was linked with an increased level of miRNA-7, for which circCDR1as is a natural sponge [38,39]. This in turn was suggested to trigger downregulation of microRNA-7-regulated genes associated with the AD, for example ubiquitin protein ligase A (*UBE2A*), an important AD therapeutic target, functionally involved in clearing toxic amyloid peptides from the patients’ brain [15]. The role of specific circRNAs has also been implicated in autoimmune diseases such as multiple sclerosis (MS), rheumatoid arthritis, systemic lupus erythematosus (SLE), lupus nephritis or primary biliary cholangitis. In most of these disorders, the proposed mechanism of circRNAs function was the regulation of expression of disease-associated proteins through interactions with miRNAs [45,46]. For example, circIBTK (hsa_circ_0077179) was found downregulated in the SLE, and linked with upregulation of miRNA-29b, which in turn induced DNA demethylation and activation of the AKT signaling pathway [90]. In the MS one of deregulated circRNAs is circGSDMB (hsa_circ_0106803) [91], whose elevated level was suggested to contribute to the alterations observed in the patients in miRNA-1275 and miRNA-149 [92]. 

### 3.1. CircRNAs in Myogenesis

Recent evidence demonstrated that circRNAs are highly expressed in skeletal muscle tissue, and global alterations in their levels were found during in vitro myogenic differentiation in either murine or human myoblasts [48,93,94]. Similarly, circRNAs abundance was found increased during neuronal differentiation [15]. These elevations are thought to be related to the high stability of circRNAs accumulating in terminally differentiated cells over time. CircRNAs levels were also changed in muscle disease conditions, suggesting important functions of these molecules in the pathogenic mechanisms [36,40]. Among circRNAs that have emerged as modulators of human myogenesis since they influenced cell differentiation and proliferation were circQKI (hsa_circ_0005328), circBNC2 (hsa_circ_0008732) and circZNF609 (hsa_circ_0000615) [48,89]. While circQKI and circBNC2 had promoting effect on differentiation, the effect of circZNF609 was the opposite. Interestingly, circZNF609 belongs to a minority of protein-coding circRNA and the RNA when ectopically expressed was shown to be translated in a splicing-dependent and cap-independent manner [48]. Interestingly, Zfp609 (mmu_circ_0001797), the mouse homolog of ZNF609, contains miRNA binding sites and was recently shown to impede the expression of myogenic transcription factors in C2C12 cells perhaps by sponging miR-194-5p [89]. 

CircRNAs involved in myogenesis have been predicted to sponge miRNAs and a number of recent studies in eukaryotes described specific competitive binding between these two classes of small RNAs. For example, circSVIL (hsa_circ_0093478), which is highly expressed during late embryonic development in chicken [95], carries four binding sites for miRNA-203. Its interaction with the miRNA (reported by in vitro assays) was found to increase the expression of *c-JUN* and *MEF2C* genes, which are miRNA-203 targets. The modulation of circSVIL expression in chicken myoblasts affected myogenic differentiation, possibly through sponging miR-203 [95]. Additional circRNAs identified as differentially expressed when compared adult to embryonic muscle tissues were shown to regulate differentiation of bovine myoblasts. For example, circFGFR4 (hsa_circ_0075146), which contains 18 putative miRNA-107 binding sites, was suggested to promote cell differentiation via targeting Wnt3a [27,96]; circFUT10 (hsa_circ_0136407) (having three putative miRNA-133a binding sites) was shown to accelerate differentiation and decrease the proliferation of myoblasts by inhibiting the miRNA activity [27,28]. On the other hand, circLMO7 (hsa_circ_0008259), which contains only one miRNA-378a-3p binding site, was shown to stimulate myoblast proliferation, impede bovine myoblast differentiation and protect myoblasts from apoptosis [96]. Altogether, these studies underline a potential regulatory role of circRNAs in the myogenesis thru the functional inactivation of miRNAs. However, interactions between endogenous miRNAs and circRNAs need to be validated since the number of miRNA binding sites identified in the circRNA sequences is very low in most of the cases reported hitherto.

### 3.2. CircRNAs in Muscle Diseases

Skeletal muscles are characterized with the highest rate of alternative splicing and alterations in canonical splicing have been associated with the pathomechanism of muscle disorders [97]. Remarkably, high expression levels of circRNAs were detected in muscle tissue from normal individuals and circRNAs dysregulation were found in muscle diseases. Recent experimental evidence showed global changes in circRNAs levels in two types of human muscular dystrophies, DMD and DM1, which are characterized by a progressive deterioration of muscle function [36,40,50,98].

DMD is triggered by frame-shifting deletions or nonsense mutations in the *DMD* gene what result in the insufficiency of the dystrophin protein [99]. Among the first circRNAs to be identified in skeletal muscles were those generated by spliced transcripts from the *DMD* [99]. Recent RNA-Seq data analysis of normal and dystrophic muscle cells revealed that DMD patient-derived cells had unique subsets of circRNAs and their expression levels differ from those of non-disease cells [48]. Interestingly, both circQKI and circBNC2 upregulated during in vitro differentiation of normal myoblasts (as described above), were downregulated in the DMD conditions. These variations were correlated with the notion that dystrophic cells have altered progression into the differentiation process [98]. Conversely, the protein-coding circZNF609, which has an inhibitory effect on differentiation and promotes myoblasts proliferation, was found at elevated level in differentiated DMD myoblasts [48]. Because circRNAs expression has been found modulated in response to cell differentiation and in disease conditions, it suggests functional involvement of these molecules in the process of muscle pathogenesis. Most recent results from another muscle-related disorder DM1 have also highlighted dysregulation of circRNAs in affected muscles when compared to healthy controls [36,40].

## 4. Dysregulation of CircRNAs in DM1

### 4.1. Global Elevation of CircRNA Levels in DM1

The most recent study which tested the hypothesis of circRNAs downregulation in DM1 known to be a burden with functional deficiency of MBNL proteins and global dysregulation of alternative splicing has brought a quite surprising discovery. In a study by Czubak et al. [40], the authors used RNA-Seq data deposited in the DMSeq database (http://www.dmseq.org/) [58], and examined circRNA levels in two types of skeletal muscles, i.e., distal (*tibialis anterior,* TA) and proximal (*quadriceps femoris, QF*) from DM1 patients and non-DM individuals. This analysis showed that among circRNAs that were significantly altered in DM1 muscles, the vast majority (i.e., 95% in QF and 87% in TA) was upregulated. This global increase was not correlated with the upregulation of expression of circRNA-corresponding linear transcripts, suggesting a different origin of circRNAs elevation. The functional association analysis of genes from which the deregulated circRNAs were generated showed that most of them were linked with the UniProt “phosphoprotein” and “alternative splicing” categories. It seems particularly interesting in the context of aberrations in alternative splicing, which are one of the molecular markers of DM1. Additionally, the term “phosphoprotein” is interesting since DM1 pathogenesis is associated with hyperphosphorylation of CUGBP1 protein [52,53], and studies have shown that the utilization of small molecule kinase inhibitors led to the alleviation of some of the molecular symptoms of DM1 including downregulation of CUGBP1 and correction of some of the mis-spliced genes [100,101].

### 4.2. Differential Expression of Individual CircRNAs in DM1

An increased level of circRNAs in DM1 skeletal muscle has also most recently been reported in another study by Voellenkle et al. [36]. The authors, who used the same DMSeq database as Czubak et al., [40] identified nine circRNAs that were significantly upregulated in DM1 muscle compared with healthy controls. Eventually, when validated in muscle biopsy samples, only four circRNAs showed an increase of expression levels in DM1 and these were circCDYL (hsa_circ_0008285), circHIPK3, circRTN4_03 (hsa_circ_0001006) and circZNF609. Interestingly, although these circRNAs were also detectable in peripheral-blood-mononuclear-cells and in the plasma samples, their levels did not differentiate DM1 and normal control samples. Elevation of expression of these four circRNAs in skeletal muscle was also found in the analysis performed by Czubak et al., [40]. These results may suggest that circRNAs upregulation is characteristics of the most severely affected tissue in DM1, i.e., skeletal muscle and may reflect one of the symptoms accompanying pathogenesis of the disease. Moreover, a substantial number of circRNAs that were identified by Czubak et al., [40] as elevated in DM1 muscles were found to be generated from multi-circRNA genes which gave rise to more than one circRNAs. Importantly, among the top MCGs were genes highly expressed in skeletal muscles and essential for their biological functions e.g., *TTN* (96 circRNA species), *nebulin* (NEB; 66 circRNA species), *triadin* (TRDN; 39 circRNA species). Interestingly, among circRNAs with decreased expression levels were circGSE1 (hsa_circ_0000722) and circBNC2, and the latter was reported to induce muscle differentiation and its downregulation was also found in DMD [48]. Thus, the reduction of expression of circBNC2 may be involved in myogenic defects which are associated with pathogenesis of both DM1 and DMD.

### 4.3. Correlation of CircRNAs Upregulation with Symptoms of DM1 Muscle Severity

**Figure 3:**
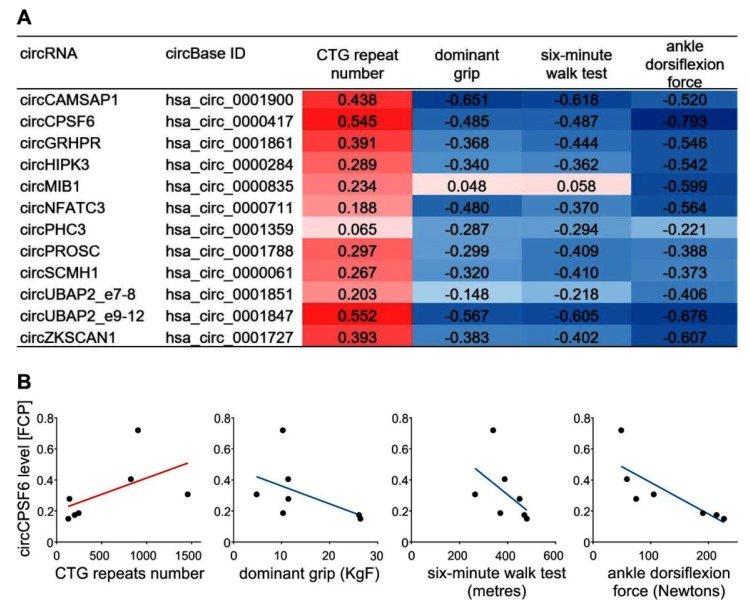
The correlation between global circRNAs levels and DM1 severity. (**A**) Listed are exemplary circRNAs which expression levels were found increased in skeletal muscles from DM1 patients [40]. Biomarkers of DM1 muscle severity, i.e., CTG repeat number, dominant grip, six-minute walk test and ankle dorsiflexion force are shown and the intensity of red and blue colors indicates, respectively, the level of positive and negative correlation. (**B**) Plots depicting the correlations of CTG repeat number, dominant grip, six-minute walk test and ankle dorsiflexion force are shown for exemplary circCPSF6 which global expression level (expressed as a fraction of circular particles, FCP) was found elevated in DM1 human muscle (for details please see Czubak et al. [40]).

Both of the recent papers that reported upregulation of circRNAs levels in DM1 pointed out its correlation with patients’ muscle severity. Czubak et al. [40] observed that for a subset of elevated circRNAs there was a negative correlation with patients’ clinical characteristics reflecting muscle weakness, i.e., dominant grip, six-minute walk test and ankle dorsiflexion force; whereas a positive correlation was found for the number of CTG repeats of the *DMPK* gene (Figure 3A,B). Similar conclusions were also derived from work by Voellenkle et al. [36], who showed that the elevated levels of circCDYL, circHIPK3, circRTN4_03, and circZNF609 were negatively correlated with skeletal muscle strength and with splicing biomarkers of disease severity, and displayed higher values in more severely affected patients.

**Figure 4:**
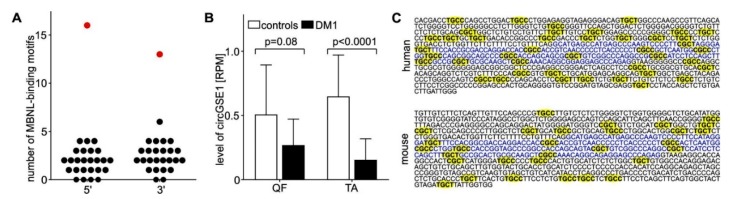
CircGSE1 as an example of MBNL-regulated RNA. (**A**) Dot plot depicting a number of potential MBNL-binding sites in introns flanking circGSE1 (red dots) and 25 randomly selected circRNAs (black dots). The regions of flanking introns embracing 300 nt upstream and 300 nt downstream from circRNA-generating exons were used to determine a number of MBNL-binding sites. (**B**) The level of circGSE1 (expressed as read per million, RPM) is decreased in two types of skeletal muscles QF and TA from DM1 patients. (**C**) Localization of potential MBNL-binding sites (highlighted in yellow) in circGSE1 exon (blue font) and in 300-nt fragments of its flanking introns (black font) is shown in both human and mouse ortholog.

Remarkably, as shown by Czubak et al. [40], the global circRNA levels were highly negatively correlated with the degree of aberrant alternative splicing. This result suggests that alterations in alternative splicing of pre-mRNAs characteristics of DM1 may also be a source of circRNAs generation and the cause of their upregulation. This notion brings up a concept of factors participating in circRNA biogenesis since the novel results by Voellenkle and Czubak question the original idea of MBNLs being universal factors of circRNAs biogenesis. These data suggest that the proteins rather than playing a global regulatory role facilitate the formation of individual species of circRNAs. This notion was supported by further results by Czubak et al. [40], who identified a subset of circRNAs whose expression was downregulated in DM1, as they had a few or none MBNL-binding motifs in their flanking intronic sequences in comparison to upregulated circRNAs (Figure 4A). One of the exceptions was circGSE1 having numerous MBNL-binding motifs, which may represent an example of MBNLs-regulated circRNAs in DM1. As depicted in Figure 4B, the expression level of circGSE1 was diminished in DM1 in comparison with normal controls. The mechanism of circGSE1 generation seems to be evolutionally conserved, since multiple MBNL-binding motifs are also present in introns flanking its mouse ortholog (Figure 4C). 

### 4.4. CircRNAs as Prospective New Biomarkers in DM1

The most recent studies, which identified upregulation of circRNAs in DM1 patients’ skeletal muscles, require more comprehensive analyses in order to determine whether circRNAs are reliable biomarkers and could be used for prognosis and as therapeutic agents and targets in DM1. A subset of circRNAs that differentiates DM1 and normal control samples have not thus far been found in any other tissue but skeletal muscle tissues. Thus, to fully determine circRNA’s potential as DM1 biomarkers in various central and peripheral tissues, a more comprehensive analysis is needed with the inclusion of more differentially expressed circRNA species.

## 5. Outlook

**Figure 5:**
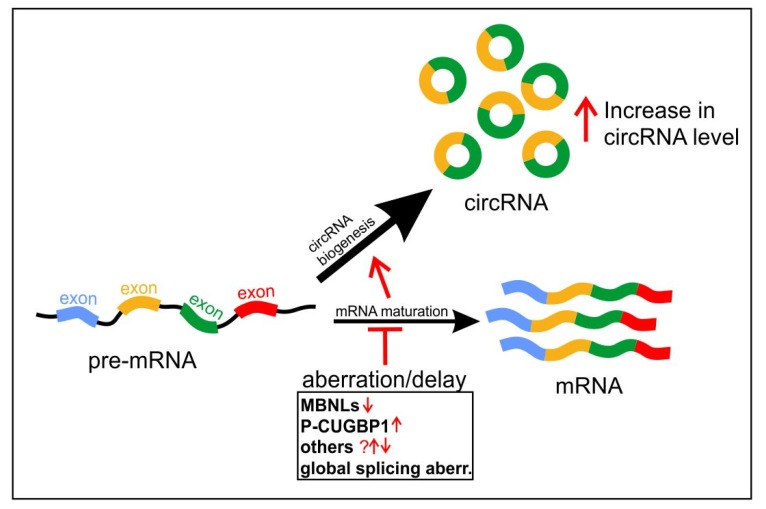
Projected mechanism of circRNAs upregulation in DM1. Global aberrations of alternative splicing are prominent features of DM1 pathogenesis. The functional depletion of MBNL proteins and changes in other proteins including splicing factors (e.g., phosphorylation of CUGBP1, P-CUGBP1) are found in DM1. Altogether, these factors may contribute to circRNAs biogenesis triggering a misbalance in the processing of pre-mRNAs into mRNAs and circRNAs. This misbalance is indicated by the size of black arrows, and by the red arrows indicating the shift toward circRNA biogenesis. The “T” bar indicate aberration/delay in mRNA formation.

Based on the current understanding, biogenesis of circRNAs might be dysregulated in conditions associated with aberrant alternative pre-mRNA splicing [102,103]. Significantly, growing experimental evidence has indicated circRNAs altered expression levels in human diseases and speculated about their role in various pathomechanisms. One of the latest discoveries has shown circRNAs upregulation in DM1, a multisystem disorder with global splicing aberrations being one of the prominent molecular biomarkers of DM1 pathomechanism [55]. A similar phenomenon has also been found in another muscular dystrophy DMD [15]. Thus, the identification and characterization of circRNAs expression in other aberrant splicing-related diseases are needed in order to decipher circRNAs role in development and progression of their pathogenesis and to help developing future therapies. Since DM1 belongs to a larger group of human neuromuscular diseases associated with mutational expansions of genetically unstable microsatellites [104,105] some elements of pathomechanism including splicing aberrations are shared between DM1 and the other diseases. Among them are myotonic dystrophy type 2 (DM2), ALS/FTD and fragile X-associated tremor/ataxia syndrome (FXTAS) [51,106]. MBNL splicing factors are, to some extent, involved also in the pathogenesis of these diseases, and thus, it would be interesting to find out whether the features of circRNAs dysregulation recently found in DM1 (Figure 5) are recapitulated in these diseases.

CircRNAs have been found to be an element of the RNA–protein network; however, their sponging activity towards miRNAs and interactions with RBPs are still poorly defined. Nevertheless, it cannot be excluded that at least some circRNAs are sequestered in DM1 in ribonucleoprotein foci. In such a scenario, their nuclear deposition in conjunction with slow turnover might contribute to the elevation of circRNAs levels observed in DM1. Only a handful of circRNAs has any function ascribed to date and it remains possible that the majority of circRNAs are accidental by-products of aberrant alternative splicing. However, the role of the global increase in circRNA levels, as well as a functional role of individual circRNAs in the pathomechanism of DM1 remains to be determined. CircRNA’s potential as novel biomarkers for prognosis and therapeutic response and as putative therapeutic tools await further investigations.
